# COPD monocytes demonstrate impaired migratory ability

**DOI:** 10.1186/s12931-017-0569-y

**Published:** 2017-05-11

**Authors:** Arjun K Ravi, Jonathan Plumb, Rosemary Gaskell, Sarah Mason, Caroline S Broome, George Booth, Matthew Catley, Jørgen Vestbo, Dave Singh

**Affiliations:** 1 NIHR Respiratory and Allergy Clinical Research Facility, Manchester Academic Health Science Centre, University Hospital South Manchester NHS Foundation Trust, University of Manchester, Manchester, UK; 2 UCB, Slough, Berkshire, UK; 3 The Medicines Evaluation Unit, Wythenshawe Hospital, The Langley Building, Southmoor Road, Wythenshawe, Greater Manchester M23 9QZ UK

**Keywords:** COPD, Monocytes, CCR5, Chemotaxis, Interleukin-6

## Abstract

**Background:**

Increased lung macrophage numbers in COPD may arise from upregulation of blood monocyte recruitment into the lungs. CCR5 is a monocyte chemokine receptor regulated by interleukin-6 (IL-6); the concentration of CCR5 ligands are known to be elevated in COPD lungs. The objective of this study was to investigate mechanisms of monocyte recruitment to the lung in COPD, including the role of CCR5 signalling.

**Methods:**

Ninety one COPD patients, 29 smokers (S) and 37 non-smokers (NS) underwent sputum induction, plasma sampling (to measure IL-6 and soluble IL-6 receptor [sIL-6R] by immunoassay), monocyte characterization (by flow cytometry) and monocyte isolation for cell migration and quantitative polymerase chain reaction studies. Lung tissue was used for immunohistochemistry.

**Results:**

Plasma IL-6 and sIL-6R levels were increased in COPD. Greater proportions of COPD CD14^++^CD16^+^ monocytes expressed CCR5 compared to controls. Monocyte stimulation with IL-6 and sIL-6R increased CCR5 gene expression. COPD monocytes demonstrated impaired migration towards sputum supernatant compared to NS (% migration, 4.4 vs 11.5, respectively; *p* < 0.05). Pulmonary microvessels showed reduced monocyte recruitment (% marginated cells) in COPD compared to NS, (9.3% vs 83.1%, respectively). The proportion of replicating Ki67^+^ alveolar macrophages was reduced in COPD compared to NS. All alveolar macrophages from COPD and S expressed the anti-apoptosis marker BCL2; this protein was not present in non-smokers or COPD ex-smokers.

**Conclusion:**

COPD monocytes show decreased migratory ability despite increased CCR5 expression. Increased COPD lung macrophage numbers may be due to delayed apoptosis.

**Electronic supplementary material:**

The online version of this article (doi:10.1186/s12931-017-0569-y) contains supplementary material, which is available to authorized users.

## Background

Monocytes can be recruited from the blood into the tissues, whereupon differentiation into macrophages may occur [1]. There are also tissue resident macrophages that replenish cell numbers by replication [2]. A recent study demonstrated the presence of phenotypically different mononuclear phagocyte cell types in healthy human lungs that either originate from the lungs (pulmonary dendritic cells and alveolar macrophages) or from blood monocytes (monocyte derived cells and tissue monocyte/macrophages) [3].

There are increased numbers of macrophages in the lungs of chronic obstructive pulmonary disease (COPD) patients [4]; these cells are involved in host defence, airway remodelling and parenchymal destruction [5]. It has been suggested that increased lung macrophage numbers in COPD are due to increased recruitment of blood monocytes [5, 6]. Alternatively, cigarette smoke exposure induces the expression of anti-apoptotic genes in macrophages [7], and increased expression of anti-apoptotic proteins has been observed in COPD macrophages [8], suggesting that delayed apoptosis is a possible cause of macrophage accumulation in COPD. Furthermore, alveolar macrophages expressing the proliferation marker Ki67 have been observed in patients with interstitial lung disease [9], but whether increased macrophage accumulation in COPD occurs by self-renewal is not understood.

Costa et al reported increased migration of COPD peripheral blood mononuclear cells towards C-X-C motif chemokine receptor 3 (CXCR3) and C-C motif chemokine receptor 5 (CCR5) ligands using single chemokines for migration experiments [6]. Such experiments, however do not reflect the complex mixture of chemoattractants present in the lungs [10–15]. Physiologically relevant complex supernatants, such as those obtained from induced sputum could be used to further investigate the migratory ability of COPD monocytes.

CCR5 is the receptor for the monocyte chemoattractant C-C motif chemokine ligand 3 (CCL3) [16]. Studies using induced sputum and bronchoalveolar lavage have shown that CCR5 ligand levels are increased in the lungs of COPD patients, suggesting a role for CCR5 signalling in the recruitment of monocytes into COPD lungs [12, 13, 16, 17]. Peripheral blood monocytes can be classified into 3 subtypes according to their expression of CD14 (LPS receptor) and CD16 (FcγRIII receptor): CD14^++^CD16^-^ (‘Classical’), CD14^+^CD16^+^ (‘Intermediate’) and CD14^-^CD16^++^ (‘Non-Classical’) [1]. Increased numbers of pro-inflammatory CD14^+^CD16^+^ monocytes are found in chronic inflammatory disease states such as rheumatoid arthritis [18]. Furthermore, CD14^+^CD16^+^ cells have the greatest surface expression of CCR5 [1, 19]. Monocyte subsets in COPD, and their expression of CCR5, have not been previously reported.

CCR5 expression is upregulated by interleukin-6 (IL-6) [20], a cytokine which trans-signals through a soluble receptor sIL-6R [21]. Plasma IL-6 levels are increased in a subset of stable COPD patients [22] and during COPD exacerbations [23]. The systemic levels of sIL-6R have not been investigated in COPD; increased systemic IL-6/sIL-6R signalling in COPD could upregulate blood monocyte CCR5 expression, thereby promoting monocyte recruitment into the lungs.

We have investigated COPD blood monocyte recruitment with two major objectives in mind. Firstly, to characterise changes in the CCL3-CCR5 axis that could facilitate monocyte recruitment in COPD; we studied CCR5 expression on peripheral blood monocytes and plasma sIL-6R levels in COPD patients compared to controls. Secondly, to further investigate the hypothesis that monocyte recruitment from the blood is increased in COPD; we studied COPD monocyte migration towards sputum supernatants and performed lung immunohistochemistry studies to evaluate monocyte migration from the pulmonary blood vessels of COPD patients compared to controls. We also performed immunohistochemistry studies to investigate an alternative mechanisms of pulmonary macrophage accumulation in COPD; namely increased replication and supressed apoptosis.

## Methods

### Subjects

COPD patients (*n* = 91), smokers (S; *n* = 29) and healthy non-smokers (HNS; *n* = 37) were recruited for blood and sputum sampling; S (with >10 pack-year smoking history) and HNS had normal lung function. COPD patients had been diagnosed according to current guidelines [24] and were also seen during acute exacerbation (diagnosed according to an increase in symptoms as described in Additional file 1). COPD (*n* = 12), S (*n* = 9) and NS (non-smokers; *n* = 6) undergoing surgical resection of suspected lung carcinoma were recruited. The research was approved by a local ethics committee; all participants provided written informed consent.

### Clinical assessment

Pre- and post- bronchodilator spirometry was performed as described in Additional file 1 [25]. COPD patients completed the St George’s respiratory questionnaire (SGRQ) and COPD Assessment Test (CAT).

### Sputum sampling

Sputum was induced and processed by the ‘two-step’ method [16]; PBS processed samples were used in the experiments reported here.

### Plasma cytokine measurement

Levels of plasma IL-6 and sIL-6R were measured using a Meso Scale Discovery immunoassay (Gaithersburg (MD–USA) (details Additional file 1).

### Monocyte chemokine receptor expression

Peripheral blood monocyte subtypes were identified by flow cytometry [1]. Proportional expression of CCR5 on CD14^++^CD16^-^, CD14^+^CD16^+^ and CD14^-^CD16^++^ monocytes was determined (details Additional file 1).

### CCL3 and Sputum supernatant induced monocyte chemotaxis

CD14^+^ monocyte isolation and chemotaxis experiments were performed as previously described (details Additional file 1) [16]. CD14^+^ monocytes did not undergo any form of cytokine stimulation prior to the chemotaxis assay.

### CD14^+^ monocyte cell culture

CD14^+^ monocytes from 6 HNS were isolated and cultured in the presence of recombinant human (rh) IL-6 (20 ng/mL, R&D Systems, Abingdon UK), rh IL-6 (20 ng/mL) + rh sIL-6R (40 ng/mL, R&D Systems) or media alone (unstimulated control). Cell lysates were harvested for gene expression analysis.

### CD14^+^ monocyte CCR5 gene expression

Quantitative polymerase chain reaction (QPCR) for determination of CCR5 gene expression was performed as previously described (details Additional file 1) [26].

### Immunohistochemistry and Immunofluorescence (IF)

See Additional file 1 for details of tissue preparation, imaging and image analysis. The immunohistochemical technique employed for detection of CD34 (endothelial cell surface glycoprotein [27]), neutrophil elastase (NE), Ki67 (cell-cycle maintaining protein) [28] and BCL-2 (B-cell CLL/lymphoma 2) (anti-apoptotic protein) (ADD REF) is detailed in Additional file 1.

Dual label immunofluorescence was performed to identify marginated ‘monocytic’ (cells apposed to the luminal endothelial surface) (CX_3_CR1^+^CD14^+^ and CX_3_CR1^+^CD16^+^) cells in the pulmonary microvasculature as described in Additional file 1.

### Statistical analysis

Normally distributed data were analysed using one way ANOVA with application of Tukey’s post-test; unpaired t-tests were used where appropriate. Non-normally distributed data were analysed using the Kruskal-Wallis test with application of Dunn’s post-test. Mann-Whitney U tests were used as appropriate. Univariate correlation analysis was performed using the Spearman Rank test. QPCR data was normally distributed and analysed using one-way ANOVA with application of Dunnett’s multiple comparisons test; details of a within subject analysis are described in Additional file 2.

Statistical analysis was performed using GraphPad Prism version 6 and IBM SPSS Statistics for Windows, Version 23.0 (released 2015). Armonk, NY.

## Results

The clinical characteristics of all study participants are stated in Table 1 (details of participants in individual experiments are described in Additional file 3: Table S1).

**Table 1:** Demographic details of study participants

	COPD (*n* = 93)	S (*n* = 29)	HNS (*n* = 39)	COPD (*n* = 12) IHC/IF	S (*n* = 9) IHC/IF	NS (*n* = 6) IHC/IF
Age	66 (7)	54.4 (7)	41.6 (15.8)	66.3 (4.6)	66.3 (6.6)	70 (5.7)
Gender (F:M)	37:58	14:15	14:25	4:8	4:5	4:2
Current smoker (n)	35	26	0	9	9	0
FEV1% predicted	60.2 (18.9)	98.1 (14.1)	109.6 (14.4)	67.3 (25.6)	91 (11.9)	108 (7.2)
FEV1/FVC (%)	52 (12.5)	75.5 (2.9)	82.5 (6.9)	67.3 (25.6)	91 (11.9)	108 (7.2)
GOLD I (%)	18.3	-	-	33.3	-	-
GOLD II (%)	50.5	-	-	25	-	-
GOLD III (%)	26.9	-	-	41.7	-	-
GOLD IV (%)	4.3	-	-	0	-	-
CAT	17.1 (7.8)					
SGRQ	39.4 (20.3)					
Smoking history (Pack Years)^a^	37 (13-122)	30.5 (12-67)	0	42 (13-126)	50 (21-66)	
ICS (%)	65	-	-			

This table shows the grouped demographic details of subjects who participated MSD plasma cytokine analysis, flow cytometric (FACS) & qPCR, chemotaxis (displayed on the left); lung immunohistochemistry/immunofluorescence (displayed on the right). Data is described by mean (SD). ^a^Data shown as median (range) *Abbreviations*: *FEV1* forced expiratory volume in 1 s, *FVC* forced vital capacity, *CAT* COPD Assessment Test and *SGRQ* St George’s Respiratory Questionnaire, *ICS* inhaled corticosteroid, *IHC* Immunohistochemistry, *IF* Immunofluorescence, *NS* non smoker

### Plasma IL-6 and sIL-6R levels

**Fig. 1:**
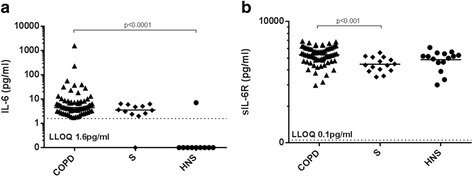
Plasma IL-6 and sIL-6R levels in COPD, S & HNS. The figure shows **a** plasma IL-6 and **b** plasma sIL-6R levels in COPD, S & HNS determined by MSD analysis. Each dot represents the value for an individual patient. The bar represents the median value. The statistical significance of differences observed was assessed by **a** The Kruskal-Wallis test with application of Dunn’s post-test and **b** ANOVA with application of Tukey’s post-test. The dashed *line* represents the lower limit of quantification (LLOQ)

Plasma IL-6 levels were significantly higher in COPD patients, (*n* = 70; median 4.5 pg/mL) compared to HNS (*n* = 15; median 0 pg/mL, *p* < 0.0001), with no other significant differences between groups (Fig. 1). Plasma sIL-6R levels were significantly higher in COPD patients compared to S (*n* = 15, medians; 5,338 pg/mL versus 4,453 pg/mL respectively, *p* < 0.001), while the comparison of COPD vs. HNS (median 4,853 pg/mL) was not significant (p = 0.3).

### Peripheral blood monocyte CCR5 expression

**Fig. 2:**
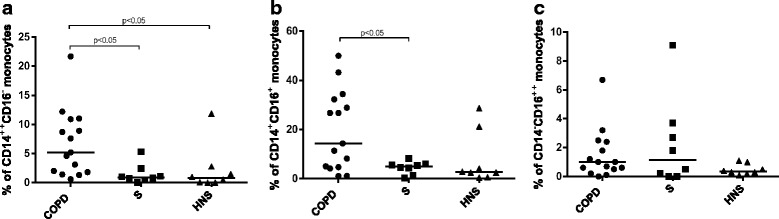
Proportions of monocyte subtypes expressing CCR5 in COPD, S & HNS. The figure shows proportions **a** CR14^++^CD16^-^, **b** CD14^+^CD16^+^ and **c** CD14^-^CD16^++^ monocytes from COPD, S and HNS expressing CCR5 determined by FACS. Each dot represents data from an individual patient; the bar represents the median value. The statistical significance of differences observed was determined by Kruskal-Wallis test with application of Dunn’s multiple comparisons test

The proportions of CD14^++^CD16^-^, CD14^+^CD16^+^ and CD14^-^CD16^++^ monocytes were similar in COPD patients (*n* = 15), S (*n* = 8) and HNS (*n* = 8) (Additional file 3: Figure S1). A significantly greater proportion of CD14^++^CD16^-^ monocytes from COPD patients expressed CCR5 compared to S or HNS; medians 5.2%, 0.9% and 0.8%, respectively (*p* < 0.05 for both comparisons; see Fig. 2). The proportion of CD14^+^CD16^+^ monocytes from COPD patients expressing CCR5 was significantly greater than S (medians 14.3% versus 4.9%; *p* < 0.05) but did not reach statistical significance compared to HNS (median 2.7%, *p* = 0.09). There were no significant differences in the proportion of CD14^-^CD16^++^ CCR5^+^ expressing cells between COPD, S and HNS; medians 1%, 1.2% & 0.4% respectively.

There was no change in the proportion of monocyte subsets or CCR5 expression at exacerbations compared to stable state (*n* = 8 COPD patients; Additional file 3: Figure S2 and Table S2).

### Regulation of monocyte CCR5 gene expression

**Fig. 3:**
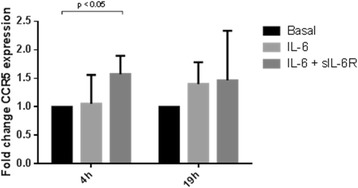
CCR5 gene expression by CD14^+^ monocytes. The figure shows changes in gene expression by CD14^+^ monocytes stimulated with IL-6 or IL-6 with sIL-6R compared with unstimulated CD14^+^ monocytes; cells were stimulated for either 4 or 19 h. The data is shown as mean (SD). The statistical significance of differences observed was determined using ANOVA with application of Dunnett’s post-test (with basal gene expression being assigned as the ‘control’)

CCR5 expression was significantly upregulated in CD14^+^ monocytes cultured for four hours in the presence of both IL-6 and sIL-6R; the mean fold change in CCR5 gene expression was 1.6 (*p* < 0.05). IL-6 stimulation alone did not upregulate CCR5 gene expression. CD14^+^ monocytes stimulated with IL-6 (solely or in conjunction with sIL-6R) for 19 h did not show alteration in CCR5 gene expression (Fig. 3 and within-subject analysis shown in Additional file 2).

### COPD CD14^+^ monocyte migration towards rhCCL3

**Fig. 4:**
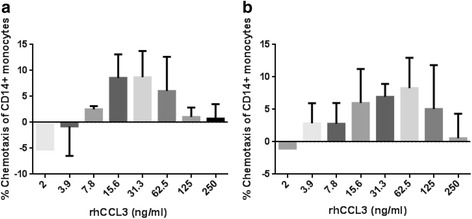
Migration of COPD CD14^+^ monocytes towards rhCCL3. This figure shows the migration of CD14^+^ monocytes from **a** COPD (*n* = 3) and **b** HNS (*n* = 3) towards a range of concentrations of rhCCL3 (250 – 2 ng/mL). Data is displayed as mean (SD)

COPD and HNS CD14^+^ monocytes showed similar migration towards rhCCL3 with a bell shaped dose-response curve observed; maximal chemotaxis levels were in the concentration range 15.6 ng/ml to 62.5 ng/ml (Fig. 4).

### CD14^+^ monocyte migration towards sputum supernatant

**Fig. 5:**
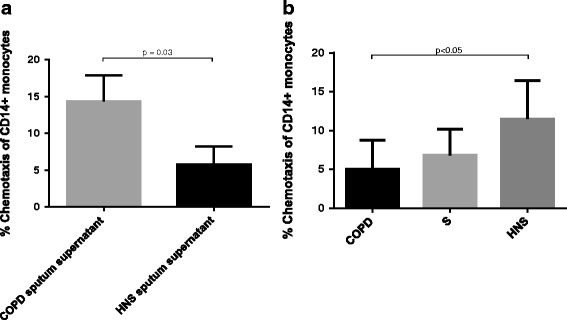
Migration of CD14^+^ monocytes towards sputum supernatant. The figure shows **a** the migration of CD14^+^ monocytes towards COPD and HNS sputum supernatant and **b** the migration of CD14^+^ monocytes isolated from COPD, S and HNS towards COPD sputum supernatant. The data is displayed as mean (SD). The statistical significance of differences observed were determined by **a** Unpaired *t* test and **b** ANOVA with application of Tukey’s multiple comparisons test. Sputum supernatant used in chemotaxis experiments was pooled from *n* = 3 donors

The migration of HNS CD14^+^ monocytes towards sputum supernatant from COPD patients and HNS was studied. A greater proportion of CD14^+^ monocytes migrated towards COPD sputum compared to HNS sputum; mean chemotaxis 14.3% versus 5.8%, respectively (*p* = 0.03) (Fig. 5). Subsequent experiments used COPD sputum supernatant as the chemoattractant. HNS CD14^+^ monocytes (*n* = 8) demonstrated significantly greater chemotaxis compared to COPD monocytes (*n* = 6); means 11.5% versus 4.4% (p < 0.05). There was a trend to significance when comparing HNS to S, (*n* = 6; mean 5.6%, *p* = 0.07).

We performed subgroup analysis based on age; monocytes of HNS >50 years of age (*n* = 3, mean age 57.7 years) with mean chemotaxis 16% demonstrated significantly greater migratory ability compared to younger HNS <50 years of age (*n* = 5, mean age 31.8 years) with mean chemotaxis 8.8 (*p* < 0.01) (Additional file 3: Figure S3).

### Marginated CX_3_CR1^+^ cells in the pulmonary microvasculature

Microvessels were identified by CD34 staining (Additional file 3: Figure S4). There were no significant differences in the size of vessels or the absolute number of marginated cells/ μm circumference observed between COPD patients (*n* = 9), S (*n* = 9) and NS (*n* = 6) (Table 2). Intravascular monocytic cells were identified by their expression of the monocyte/macrophage surface marker CX_3_CR1. The median number of marginated CX_3_CR1^+^ cells per μm vessel circumference were significantly greater in NS (83.9 x10^-4^/μm) and S (31.5 x10^-4^/μm) compared to COPD (1.5 × 10^-4^/μm;p < 0.01 and *p* < 0.05 respectively). The median percentage of marginated cells that expressed CX_3_CR1^+^ was significantly greater in HNS (83.1%) and S (46%) compared to COPD (9.3%; *p* < 0.001 and *p* < 0.05 respectively) (Table 2 & Fig. 5). A large number of the CX_3_CR1^-^ marginated cells in COPD patients were neutrophils based on their appearance and nuclear morphology, which was confirmed by NE staining (Additional file 3: Figure S5).

**Table 2:** Marginated CX_3_CR1^+^ cells per unit vessel circumference

	COPD (*n* = 9)	S (*n* = 9)	NS (*n* = 6)	*p* value
Vessel circumference (μm)	722 (206.9)	902 (275.2)	766 (227.2)	ns
Marginated cells/vessel circumference (10^-4^/μm)	10 (8)	9 (9)	20 (20)	ns
Marginated CX_3_CR1^+^ (%)	3.1 (0-35.8)	48.3 (10-73.7)	85.8 (50-100)	0.0002
CX_3_CR1^+^/vessel circumference (10^-4^/μm)	1 (0-80)	30 (5-200)	100 (20-1000)	0.005
CX_3_CR1^+^CD14^+^(Marginated CX_3_CR1^+^ cells %)	0 (0-3.6)	26.7 (0-49.2)	73.4 (6.7-93.8)	0.0008
CX_3_CR1^+^CD14^+^/vessel circumference (10^-4^/μm)	0 (0-3)	20 (0-100)	50 (2-400)	0.002
CX_3_CR1^+^CD16^+^(Marginated CX_3_CR1^+^ cells %)	0 (0-29.3)	17.3 (0-60.4)	43 (20-69.9)	0.001
CX_3_CR1^+^CD16^+^/vessel circumference (10^-4^/μm)	0 (0-100)	10 (0-100)	100 (10-400)	0.02

This table shows CX_3_CR1^+^ cells as a percentage of all marginated cells as well the absolute number of CX_3_CR1^+^ cells per μm of vessel circumference (*10^-^4/μm). Data is displayed as median (range). The statistical significance of differences observed was obtained using Kruskal-Wallis test with application of Dunn’s multiple comparisons test. Vessel circumference and marginated cells/μm vessel circumference are displayed as mean (SD); the statistical significance of differences observed was assessed using ANOVA with application of Tukey’s post-test

**Fig. 6:**
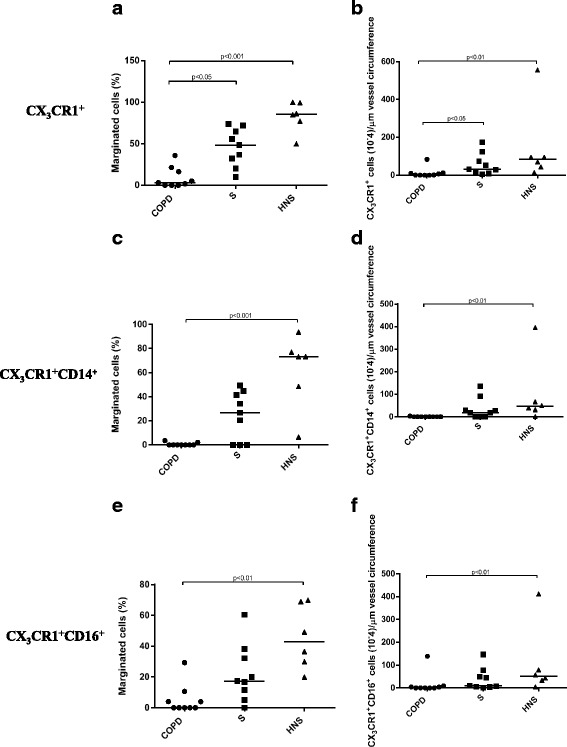
Marginated intravascular CX_3_CR1^+^, CX_3_CR1^+^CD14^+^ and CX_3_CR1^+^CD16^+^ cells. This figure shows the proportions of marginated CX_3_CR1^+^ (5**a** & 5**b**), CX_3_CR1^+^CD14^+^ (5**c** and 5**d**) and CX_3_CR1^+^CD16^+^ (5**e** & 5**f**) cells. The bar represents the median; each data point represents the value for an individual patient. The statistical significance of differences observed was determined using Kruskal-Wallis test with application of Dunn’s multiple comparisons test

**Fig. 7:**
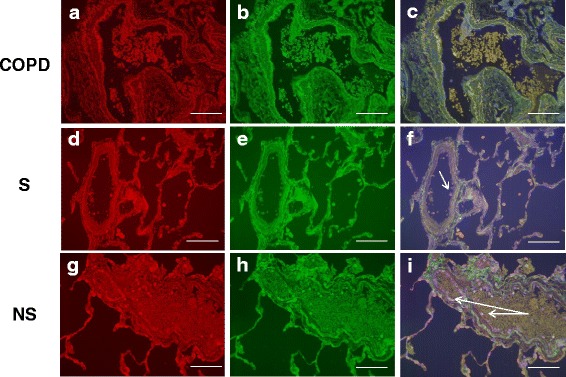
CX_3_CR1 CD14 immunofluorescent staining of the pulmonary microvasculature of COPD, S and NS. These figures are representative images for dual label immunofluorescent detection of CD14 by CX_3_CR1^+^ monocytes marginated within pulmonary microvessels in human lung tissue. Representative images 6**a**-**c**) *n* = 9 COPD, 6**d**-**f**) *n* = 9 S and 6 **g**-**i**) *n* = 6 NS. Cell nuclei were counterstained with 4’,6-diamidino-2-phenylindole (*blue*). CX_3_CR1^+^ cells were identified using an Alexa-568 conjugated donkey anti-rabbit secondary antibody (*red* 6**a**, 6**d**, 6 **g**). CD14^+^ cells were labelled with a biotinylated rabbit anti-goat secondary antibody and detected using Streptavidin Dylight 488 (*green* 6**b**, 6**e**, 6 **h**). Composite images are shown (6**c**, 6**f**, 6**i**). *Green*/*yellow* autofluorescence is caused by intrinsically fluorescent tissue components such as elastic fibres and erythrocytes. Autofluorescence can be distinguished from positive fluorescence by forming a composite image of the *red*, *green* and *blue* channels. Autofluorescence is visible in all three channels and so appears as an amalgamation of the three colours. Positive fluorescence is visible in one channel only and thus appears as the pure colour. Singly labelled cells appear in the composite image as either being *red* or *green*, dual labelled cells appear *yellow*. Examples of immunoreactive cells are indicated by *arrows* (images taken using X20 objective lens). The white scale bar represents a length of 75 μm

**Fig. 8:**
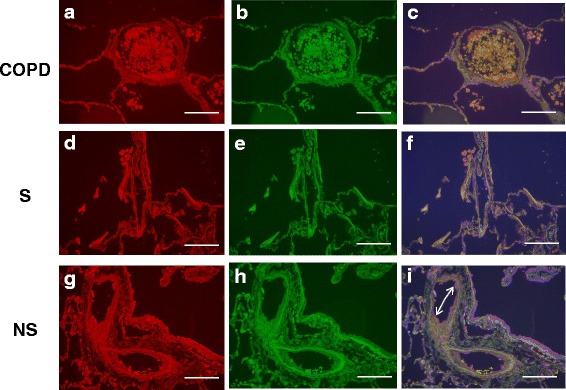
CX_3_CR1 CD16 Immunofluorescent staining of the pulmonary microvasculature of COPD, S and NS These figures are representative images for dual label immunofluorescent detection of CD16 by CX_3_CR1^+^ monocytes marginated within pulmonary microvessels in human lung tissue. Representative images 7**a**-**c**) 9 COPD, 7**d**-**f**) 9 S and 7 **g**-**i**) 6 NS. Cell nuclei were counterstained with 4’,6-diamidino-2-phenylindole (*blue*). CX_3_CR1^+^ cells were identified using an Alexa-568 conjugated donkey anti-rabbit secondary antibody *red* 7**a**, 7**d**, 7 **g**). CD16^+^ cells were labelled with a biotinylated horse anti-mouse secondary antibody and detected using Streptavidin Dylight 488 (*green* 7**b**, 7**e**, 7 **h**). Composite images are shown (7**c**, 7**f**, 7**i**). Autofluorescence is visible in all three channels and so appears as an amalgamation of the three colours. Positive fluorescence is visible in one channel only and thus appears as the pure colour. Singly labelled cells appear in the composite image as either being *red* or *green*, dual labelled cells appear *yellow*. Examples of immunoreactive cells are indicated by *arrows* (images taken using X20 objective lens). The *white scale* bar represents a length of 75 μm

The CX_3_CR1^+^ marginated cell population was composed of CX_3_CR1^+^CD14^+^ and CX_3_CR1^+^CD16^+^ cells. The absolute number and percentage of marginated CX_3_CR1^+^CD14^+^ cells per μm vessel circumference was significantly reduced in COPD patients compared to both S and NS (Table 2, Figs. 6 and 7). Similar results were obtained for marginated CX_3_CR1^+^CD16^+^ cells in COPD patients compared to S and NS (Table 2, Figs. 6 and 8). Negative control immunofluorescence images are displayed in Additional file 3: Figure S6.

### Alveolar macrophage Ki67 and BCL2 expression

**Fig. 9:**
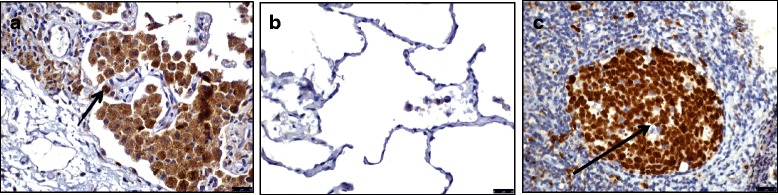
Alveolar macrophage Ki67 expression. This figure shows immunohistochemical detection of Ki67 in lung resection specimens of COPD, S and NS. Representative images of *n* = 9 COPD, *n* = 9 S and *n* = 6 NS. Positively labelling cells were visualised using DAB. Immunoreactive cells (*brown nuclei*) are indicated with an *arrow*. 8**a**) Ki67 labelled lung resection specimen 8**b**) negative control 8**c**) tonsilar tissue (positive control). Images taken using an X40 objective lens

COPD patients (*n* = 9) and S (*n* = 9) had significantly greater mean numbers of alveolar macrophages (59.2/mm^2^ and 64.7/mm^2^ respectively) compared to NS (*n* = 6; 20.5/mm^2^, *p* < 0.05 and *p* < 0.01 respectively). The percentage of Ki67^+^ alveolar macrophages was low, and greater in NS (mean 2%) compared to COPD patients (mean 0.9%, *p* < 0.05 vs NS) (Fig. 9).

**Fig. 10:**
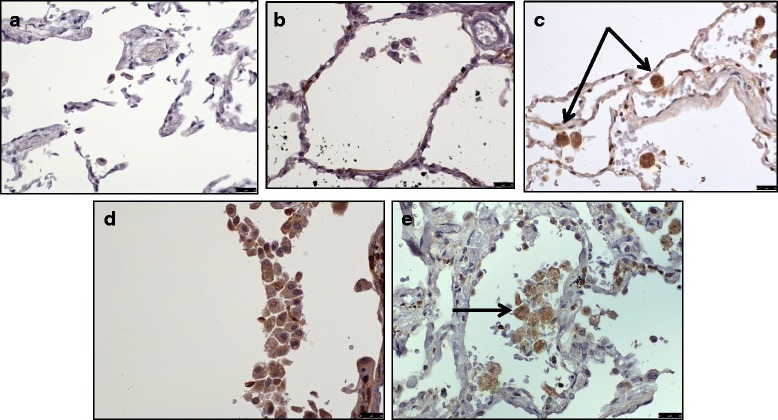
BCL2 expression of alveolar macrophages. This figure shows immunohistochemical detection of BCL-2 in lung resection specimens of COPD, S and NS. Representative images of *n* = 9 COPD, *n* = 3 COPD former smoker, *n* = 9 S and *n* = 6 NS. Positively labelling cells were visualised using DAB (immunoreactive cells have *brown* cytoplasm and are indicated with an arrow). 9**a**) negative control, 9**b**) NS, 9**c**) S, 9**d**) COPD former smoker and 9**e**) COPD current smoker. Images taken using an X 40 objective lens

BCL2 was expressed by all COPD current smokers (*n* = 9) and S (*n* = 9) alveolar macrophages, however BCL2 was absent in the alveolar macrophages of NS (*n* = 6) and COPD former smokers (*n* = 3) (Fig. 10).

## Discussion

We observed increased CCR5 expression on COPD blood monocytes. Increased plasma levels of sIL-6R may play a role in this observation, as IL-6 with sIL-6R upregulated CCR5 gene expression in monocytes. However, this increase in COPD monocyte CCR5 expression did not confer a greater migratory ability; chemotaxis experiments showed impairment of the migratory ability of COPD peripheral blood monocytes. This impaired migration was confirmed by examining monocyte margination from blood vessels in the lungs. We have therefore found no evidence to support the theory that increased monocyte recruitment is responsible for increased lung macrophage accumulation in COPD.

We investigated alternative mechanisms that could contribute to lung macrophage accumulation in COPD. There were low levels of alveolar macrophage self-renewal in COPD, suggesting that this mechanism does not contribute to the macrophage accumulation in COPD. Current smokers, with and without COPD, had increased expression of the anti-apoptotic marker BCL-2 in alveolar macrophages. These findings suggest a mechanism by which macrophage accumulation can occur in smokers and COPD patients.

The mechanisms for impaired COPD monocyte migration may be related to systemic oxidative stress, which is increased in S [29] and COPD [29, 30]. It is known that monocytes from S display migratory impairment [31]; this phenomenon can be induced in HNS monocytes after exposure to radical and non-radical oxidants [31]. We observed a trend toward significant reduction in monocyte migration when comparing S and HNS. Inflammatory cell chemotaxis may diminish with increasing age [32], however a subgroup analysis of our data in HNS monocytes failed to show such an association.

A recent study showed that lymphocytes and monocytes interact to facilitate peripheral blood mononuclear cell (PBMC) chemotaxis towards CXCR3 and CCR5 ligands, but that isolated COPD monocytes had similar chemotaxis ability to controls [6]. We used a more complex chemotactic system with induced sputum supernatant and isolated monocytes. We have previously shown that CCL3 (a ligand for CCR5) levels are increased in COPD sputum, and that CCL3 and CCR5 blockade reduces monocyte chemotaxis to COPD sputum supernatant [16]; we now demonstrate that exposure to rhCCL3, induces migration of COPD CD14^+^ monocytes. We also observed increased CCR5 expression on CD14^++^CD16^-^ and CD14^+^CD16^+^ COPD blood monocytes, so it was perhaps surprising that COPD monocytes displayed impaired chemotaxis. It was therefore important that we evaluated monocyte migration into the lungs by a different method to further investigate this observation.

C-X3-C motif chemokine receptor 1 (CX_3_CR1) is a widely used monocyte/macrophage marker [1, 33]. We found reduced margination of CX_3_CR1^+^ cells in COPD compared to NS. The vascular endothelium of pulmonary vessels in S and COPD expresses increased levels of adhesion molecules such as E-selectin, P selectin, ICAM-1, ICAM-2 and VCAM-1 [34]. It is therefore unlikely that the reduced margination observed in COPD resulted from attenuated endothelial adhesion molecule expression. The reduced margination was observed for both CX_3_CR1^+^CD14^+^ and CX_3_CR1^+^CD16^+^ cells in COPD patients.

Attenuated monocyte migration ability in COPD raises questions regarding the mechanisms of increased lung macrophage numbers in COPD patients. Desch et al. recently described a “tissue monocyte” population in healthy human lungs that resembles monocytes, but expresses cell surface markers found in alveolar macrophages [3]. Populations described as “monocyte derived cells” were also identified, which are thought to be monocytes that change phenotype and acquire new cell surface markers including CD206 when recruited from the blood into the lungs, as previously reported in mice [35]. Our results support the concept that monocytes can be recruited into the lungs, as we observed marginated monocytes in control lung samples. However, monocyte margination was reduced in COPD patients, indicating that increased lung macrophage numbers in COPD are not simply due to excessive blood monocyte recruitment.

Murine studies have shown that lung macrophages can be replenished by self-renewing, locally-derived progenitor cells [2, 36]. Human studies have also shown that resident lung mononuclear phagocyte populations can express the cell-cycle maintaining protein Ki67, suggesting that these cells are engaged in self-renewal [37, 38]. We observed low levels of Ki67 expression amongst alveolar macrophages, suggesting that a very limited proportion of these cells were actively undergoing self-renewal. Simian studies have suggested that the turnover of these cells is indeed very low whereas the turnover of interstitial lung macrophages is high [39].

It has been reported that COPD lung macrophages express increased levels of a protein known as ‘Apoptosis inhibitor of macrophage (AIM)’ which is associated with delayed apoptosis [8]. A different study reported that alveolar macrophages from smokers displayed higher levels of anti-apoptotic proteins including Bcl-_xL_ [7]. These previous studies support the hypothesis that delayed apoptosis, caused by cigarette smoking, contributes to macrophage accumulation in COPD. We observed that the anti-apoptotic protein BCL-2 was only present in current smokers, with and without COPD. Our findings support a role for current smoking in prolonging alveolar macrophage lifespan. We speculate that the increased alveolar macrophage numbers due to active smoking does not return to normal after smoking cessation, as the years of chronic cigarette smoking causing delayed apoptosis have permanently altered the homeostasis of lung macrophage numbers. We also note that Kojima et al found increased AIM expression in COPD alveolar macrophages compared to both smoking and non-smoking controls, implicating this particular anti-apoptotic protein in COPD specific mechanisms [8]. Overall, these previous findings and our current observations indicate mechanisms of delayed apoptosis that can occur in macrophages from current smokers or COPD patients.

In keeping with previously published studies, we observed increased plasma IL-6 levels in stable COPD patients [22]. We also observed significantly increased plasma sIL-6R levels in COPD patients compared to S. IL-6 signals through either membrane-bound IL-6R or sIL-6R [21]. Increased sIL-6R levels may amplify the effects of IL-6 [40]. CCR5 gene expression in microglial cells is upregulated following culture with IL-6 [20]; in the same study IL-6 stimulation caused a numerical, but not statistically significant, increase in CCR5 gene expression in healthy blood monocytes [20]. We also observed a small increase in CCR5 expression with IL-6 alone, but significant induction was achieved when sIL-6R was also present, suggesting an important role for IL-6 trans-signalling in the regulation of CCR5 expression. An alternative mechanism for CCR5 upregulation in monocytes is the effects of reactive oxygen species exposure, which can upregulate CCR5 expression [41, 42].

Increased sIL-6R levels may promote inflammatory activity in COPD by enhancing the effects of IL-6 through trans-signalling. IL-6 is involved in the polarization of naïve CD4^+^ T lymphocytes towards the pro-inflammatory Th17 effector phenotype [43]; furthermore, IL-6 suppresses apoptosis of both innate and adaptive immune cells resulting in their persistence at foci of inflammation [44, 45].

CD14^+^CD16^+^ monocytes are potent secretors of IL-1, IL-6 and TNF-α [1], and expanded CD14^+^CD16^+^ monocyte populations are found in inflammatory disease states including atherosclerosis [46], obesity [47] and rheumatoid arthritis [48]. We did not observe any change in monocyte subsets in COPD patients compared to controls. Furthermore, there was no change in cellular subpopulations during exacerbations, indicating no dysregulation of CD14^+^CD16^+^ cells in COPD.

We elected to use sputum for chemotaxis experiments as there are increased levels of monocyte chemoattractants in the sputum supernatants of COPD patients compared to controls [14, 16]. Furthermore, the total number of macrophages in sputum is increased in COPD patients [14, 16]. Bronchoalveolar lavage supernatants are an alternative for chemotaxis experiments, representing a different lung compartment where macrophages numbers are increased. However, this is more invasive and the installation of saline may cause excessive dilution.

## Conclusion

In conclusion, we have demonstrated that COPD monocytes show decreased migratory ability despite augmented expression of CCR5. These findings indicate that increased lung macrophage numbers in COPD lungs are not be due to excessive blood monocyte recruitment or alveolar macrophage self-renewal; suppressed apoptosis may be a factor leading to macrophage accumulation in the lungs of COPD patients.

## Additional files

Additional file 1: Details of methodology (pulmonary function, plasma separation & cytokine measurement, flow cytometric monocyte characterisation, CD14^+^ monocyte isolation, sputum supernatant induced monocyte chemotaxis, CD14^+^ monocyte chemokine receptor gene expression, Quantitative polymerase chain reaction, Immunohistochemistry and Immunofluorescence. (DOCX 34 kb)

Additional file 2: Details of ‘Within-subject analysis’ (Generalised Estimation Equation analysis) from CCR5 gene expression study (DOCX 15 kb)

Additional file 3: Details of legends for additional Figures S1-S6. **Table S1.** demographic details of patients from cytokine analysis, flow cytometry, chemotaxis and gene expression studies. **Table S2.** proportions of peripheral blood monocyte subtypes (CD14^++^CD16^-^, CD14^+^CD16^+^ & CD14^-^CD16^++^) in stable and exacerbating COPD patients. **Figure S1.** proportions of monocyte sub-populations in the blood of COPD, S & HNS displayed as a graph. **Figure S2.** changes in the CCR5 expression by monocyte sub populations during COPD exacerbations displayed as a graph. **Figure S3.** Eeffect of age on CD14^+^ monocyte migration displayed as a graph. **Figure S4.** CD34 expression of pulmonary endothelial cells displayed as an immunohistochemistry image. **Figure S5.** neutrophils in the pulmonary microvasculature of COPD patients displayed as an immunohistochemistry image. **Figure S6.** Double negative immunofluorescent image of tonsilar tissue stained using an immunofluorescence protocol with omission of CX_3_CR1 and CD14 primary antibodies. (ZIP 2021 kb)

## Abbreviations

AIM: Apoptosis inhibitor of macrophage
ANOVA: Analysis of variance
BCL2: B-cell CLL/lymphoma 2
BCL-_XL_: B cell lymphoma extra large
CAT: COPD assessment test
CCL: C-C motif chemokine ligand
CCR: C-C motif chemokine receptor
CD: Cluster of differentiation
COPD: Chronic obstructive pulmonary disease
C-X3-CR: CX_3_C chemokine receptor
CXCR: C-X-C motif chemokine receptor
HNS: Healthy non-smoker
ICAM: Intercellular adhesion molecule
IF: Immunofluorescence
IHC: Immunohistochemistry
IL: Interleukin
NE: Neutrophil elastase
NS: Non-smoker
PBMC: Peripheral blood mononuclear cell
QPCR: Quantitative polymerase chain reaction
rh: Recombinant human
S: Smoker
SGRQ: St George’s Respiratory Questionnaire
sIL-6R: Soluble Interleukin-6 Receptor
TNF: Tumour necrosis factor
VCAM: Vascular cell adhesion molecule

## References

[1] Cros J, Cagnard N, Woollard K, Patey N, Zhang S-Y, Senechal B, Puel A, Biswas SK, Moshous D, Picard C, Jais J-P, D'Cruz D, Casanova J-L, Trouillet C, Geissmann F (2010). Human CD14^dim^ Monocytes Patrol and Sense Nucleic acids and viruses via TLR7 and TLR8 receptors. Immunity.

[2] Yona S, Kim K-W, Wolf Y, Mildner A, Varol D, Breker M, Strauss-Ayali D, Viukov S, Guilliams M, Misharin A, Hume DA, Perlman H, Mailssen B, Zelzer E, Jung S (2013). Fate mapping reveals origins and dynamics of monocytes and tissue macrophages under homeostasis. Immunity.

[3] Desch AN, Gibbings SL, Goyal R, Kolde R, Bednarek J, Bruno T, Slanksy JE, Jacobelli J, Mason R, Ito Y, Messier E, Randolph GJ, Prabagar M, Atif SM, Segura E, Xavier RJ, Bratton DL, Janssen WJ, Henson PM, Jakubzick C (2016). Flow cytometric analysis of mononuclear phagocytes in nondiseased human lung and lung-draining lymph nodes. Am J Respir Crit Care Med.

[4] Hogg JC, Chu F, Utokaparch S, Woods R, Elliott WM, Buzatu L, Cherniak RM, Rogers RM, Sciurba FC, Coxson HO, Pare PD (2004). The Nature of Small Airway Obstruction in Chronic Obstructive Pulmonary Disease. N Engl J Med.

[5] Barnes PJ (2000). Chronic obstructive pulmonary disease. N Engl J Med.

[6] Costa C, Traves SL, Tudhope SJ, Fenwick PS, Belchamber KBR, Russel REK, Barnes PJ, Donnelly LE (2016). Enhanced monocyte migration to CXCR3 and CCR5 chemokines in COPD. Eur Respir J.

[7] Tomita K, Caramori G, Lim S, Ito K, Hanazawa T, Oates T, Chiselita I, Jazrawi E, Chung KF, Barnes PJ, Adcock IM (2002). Increased p21CIP1/WAF1 and B Cell Lymphoma Leukemia-XL expression and reduced apoptosis in alveolar macrophages from smokers. Am J Respir Crit Care Med.

[8] Kojima J, Araya J, Hara H, Ito S, Takasaka N, Kobayashi K, Fujii S, Tsurushige C, Numata T, Ishikawa T, Shimizu K, Kawaishi M, Saito K, Kamiya N, Hirano J, Odaka M, Morikawa T, Hano H, Arai S, Miyazaki T, Kaneko Y, Nakayama K, Kuwano K (2013). Apoptosis inhibitor of macrophage (AIM) expression in alveolar macrophages in COPD. Respir Res.

[9] Wahlström J, Berlin M, Sköld CM, Wigzell H, Eklund A, Grunewald J (1999). Phenotypic analysis of lymphocytes and monocytes/macrophages in peripheral blood and bronchoalveolar lavage fluid from patients with pulmonary sarcoidosis. Thorax.

[10] Aaron SD, Vandemheen KL, Ramsay T, Zhang C, Avnur Z, Nikolcheva T, Quinn A. Multi-analyte profiling and variability of inflammatory markers in blood and induced sputum in patients with stable COPD. Respir Res [serial online] 2010; vol. 11. Available from: https://respiratory-research.biomedcentral.com/articles/10.1186/1465-9921-11-41 10.1186/1465-9921-11-41 PMC2874769 20412595

[11] Bafadhel M, McCormick M, Saha S, McKenna S, Shelley M, Hargadon B, Mistry V, Reid C, Brightling CE (2012). Profiling of sputum inflammatory mediators in asthma and chronic obstructive pulmonary disease. Respiration.

[12] Capelli A, Di Stefano A, Gnemmi I, Balbo P, Cerutti CG, Balbi B, Lusuardi M, Donner CF (1999). Increased MCP-1 and MIP1β in bronchoalveolar lavage fluid of chronic bronchitics. Eur Respir J.

[13] Costa C, Rufino R, Traves SL, Silva JR LE, Barnes PJ, Donnelly LE (2008). CXCR3 and CCR5 chemokines in Induced Sputum from patients with COPD. Chest.

[14] Traves SL, Culpit SV, Russell REK, Barnes PJ, Donnelly LE (2002). Increased levels of the chemokines Gro-α and MCP1 in sputum samples from patients with COPD. Thorax.

[15] Saetta M, Mariani M, Panina-Bordignon P, Turato G, Buonsanti C, Baraldo S, Bellettato CM, Papi A, Corbetta L, Zuin R, Sinigaglia F, Fabbri L (2002). Increased expression of the chemokine receptor CXCR3 and its ligand CXCL10 in peripheral airways of smokers with chronic obstructive pulmonary disease. Am J Respir Crit Care Med.

[16] Ravi AK, Khurana S, Lemon J, Plumb J, Booth G, Healy L, Catley M, Vestbo J, Singh D (2014). Increased levels of soluble interleukin-6 receptor and CCL3 in COPD sputum. Respir Res.

[17] Smyth LJC, Starkey C, Gordon FS, Vestbo J, Singh D (2008). CD8 chemokine receptors in chronic obstructive pulmonary disease. Clin Exp Immunol.

[18] Kawanaka N, Yamamura M, Aita T, Morita Y, Okamoto A, Kawashima M, Iwashi M, Ueno A, Ohmoto Y, Makino H (2002). CD14+, CD16+ blood monocytes and joint inflammation in rheumatoid arthritis. Arthritis Rheum.

[19] Weber C, Belge KU, von Hundelschausen P, Drauda G, Steppich B, Mack M, Frankenberger M, Weber KSC, Ziegler-Heitbrock HW (2000). Differential chemokine receptor expression and function in human monocyte subpopulations. J Leukoc Biol.

[20] Wang J, Crawford K, Yuan M, Wang H, Gorry PR, Gabuzda D (2002). Regulation of CC chemokine receptor 5 and CD4 expression and human immunodeficiecy virus Type 1 replication in human macrophages and microglia by T helper Type 2 Cytokines. J Infect Dis.

[21] Fielding CA, McLoughlin RM, Mcleod L, Colmont CS, Najdovska M, Grail D, Ernst M, Jones SA, Topley N, Jenkins BJ (2008). IL-6 regulates neutrophils trafficking during acute inflammation via STAT3. J Immunol.

[22] Agusti A, Edwards LD, Rennard SI, Macnee W, Tal-Singer R, Miller BE, Vestbo J, Lomas DA, Calverley PMA, Wouters E, Crim C, Yates JC, Silverman EK, Coxson HO, Bakke P, Mayer RJ, Celli B, for-the-Evaluation-of-COPD-Longitudinally-to-Identify-Predictive-Surrogate-Endpoints-(ECLIPSE)-Investigators (2012). Persistent Systemic Inflammation is Associated with Poor Clinical Outcomes in COPD: A Novel Phenotype. PLoS One.

[23] Hurst JR, Perera WR, Wilkinson TMA, Donaldson GC, Wedzicha JA (2006). Systemic and upper and lower airway inflammation at exacerbation of chronic obstructive pulmonary disease. Am J Respir Crit Care Med.

[24] Vogelmeier CF, Criner GJ, Martinez FJ, Anzueto A, Barnes PJ, Bourbeau J, Celli BR, Chen R, Decramer M, Fabbri LM, Frith PA, Halpin DMG, Victorina López Varela M, Nishimura M, Roche N, Rodriguez-Rosin R, Sin DD, Singh D, Stockley RA, Vestbo J, Wedzicha JA, Agusti A (2017). Global strategy for the diagnosis, management and prevention of chronic obstructive lung disease 2017 Report - GOLD executive summary. Am J Respir Crit Care Med.

[25] Wanger J, Clausen JL, Coates A, Pedersen O, Brusasco V, Burgos F, Casaburi R, Crapo R, Enright P, van der Grinten CPM, Gustafsson P, Hankinson J, Jensen R, Johnson D, MacIntyre N, McKay R, Miller MR, Navjas D, Pellegrino R, Viegi G (2005). Standardization of the measurement of lung volumes. Eur Respir J.

[26] Lea S, Plumb J, Metcalfe H, Spicer D, Woodman P, Fox JC, Singh D (2014). The effect of peroxisome proliferator-activated receptor-Ɣ ligands on in vitro and in vivo models of COPD. Eur Respir J.

[27] Pusztazeri MP, Seelentag W, Bosman FT (2005). Immunohistochemical expression of endothelial markers CD31, CD34, von Willebrand Factor, and Fli-1 in normal human tissues. J Histochem Cytochem.

[28] Braun N, Papadopolous T, Müller-Hermelink H (1988). Cell cycle dependent distribution of the proliferation-associated Ki-67 antigen in human embryonic lung cells. Virchows Arch B, Cell pathol incl mol pathol.

[29] Rahman I, Morrison D, Donaldson K, Macnee W (1996). Systemic oxidative stress in asthma, COPD and smokers. Am J Respir Crit Care Med.

[30] Couillard A, Koechlin C, Cristol JP, Varray A, Prefaut C (2002). Evidence of local exercise induced systemic oxidative stress in chronic obstructive pulmonary disease. Eur Respir J.

[31] Stadler N, Eggermann J, Vöö S, Kranz A, Waltenberger J (2007). Smoking-induced monocyte dysfunction is reversed by vitamin C supplementation in vivo. Arterioscler, Thromb Vasc Biol.

[32] Wenisch C, Patruta S, Daxböck F, Krause R, Hörl W (2000). Effect of age on human neutrophil function. J Leukoc Biol.

[33] McComb JG, Ranganathan M, Liu XH, Pilewski JM, Ray P, Watkins SC, Choi AMK, Lee JS (2008). CX3CL1 Up-regulation is associated with recruitment of CX3CR1+ Mononuclear Phagocytes and T lymphocytes in the lungs during cigarette smoke-induced emphysema. Am J Pathol.

[34] González S, Hards J, van Eeden S, Hogg JC (1996). The expression of adhesion molecules in cigarette smoke-induced airways obstruction. Eur Respir J.

[35] Jakubzick C, Gautier EL, Gibbings SL, Sojka DK, Schlitzer A, Johnson TE, Ivanov S, Duan Q, Bala S, Condon T, van Rooijen A, Grainger JR, Belkaid Y, Ma'ayan A, Riches DWH, Yokoyama WM, Ginhoux F, Henson PM, Randolph GJ (2013). Minimal differentiation of classical monocytes as they survey steady-state tissues and transport antigen to lymph nodes. Immunity.

[36] Perdiguero E-G, Klapproth K, Schultz C, Busch K, Azzoni E, Crozet L, Garner H, Trouillet C, de Bruijn M, Geissmann F, Rodewald H-R (2015). Tissue-resident macrophages originate from yolk-sac-derived erythro-myeloid progenitors. Nature.

[37] Brittan M, Barr LC, Anderson N, Conway Morris A, Duffin R, Marwick JA, Rossi F, Johnson S, Dhaliwal K, Hirani N, Rossi AG, Simpson AJ (2014). Functional characterisation of human pulmonary monocyte-like cells in lipopolysaccharide-mediated acute lung inflammation. J Inflamm.

[38] Eguíluz-Gracia I, Schultz HHL, Sikkeland LIB, Danilova E, Holm AM, Pronk CJH, Agace WW, Iversen M, Andersen C, Jahnsen FL, Baekkevold ES (2016). Long-term persistence of human donor alveolar macrophages in lung transplant recipients. Thorax.

[39] Cai Y, Sugimoto C, Arainga M, Alvarez X, Didier ES, Kuroda MJ (2014). In vivo characterization of alveolar and interstitial lung macrophages in rhesus macaques: implications for understanding lung disease in humans. J Immunol.

[40] Hurst SM, Wilkinson TS, McLoughlin RM, Jones S, Horiuchi S, Yamamoto N, Fuller GM, Topley N, Jones SA (2001). IL-6 and its soluble receptor orchestrate a temporal switch in the pattern of leucocyte recruitment seen during acute inflammation. Immunity.

[41] Saccani A, Saccani S, Orlando S, Sironi M, Ghezzi P, Mantovani A, Sica A (2000). Redox regulation of chemokine receptor expression. Proc Natl Acad Sci.

[42] Lehoux G, Le Gouill C, Stankova J, Rola-Pleszcynski M (2003). Upregulation of the expression of CCR5 by hydrogen peroxide in human monocytes. Med Inflamm.

[43] Benwell R, Lee DR (2009). Essential and synergistic Roles of IL-1 and IL-6 in human Th17 differentiation directed by TLR ligand activated dendritic cells. Clin Immunol.

[44] Asensi V, Valle E, Meana A, Fierer J, Celada A, Alvarez V, Paz J, Coto E, Carton JA, Maradona JA, Dieguez A, Sarasua J, Ocana M, Arribas JM (2004). In vivo Interleukin-6 protects neutrophils from apoptosis in osteomyelitis. Infect Immun.

[45] Atreya R, Finotto S, Mullberg J, Jostock T, Wirtz S, Schutz M, Bartsch B, Holtmann M, Becker C, Strand D, Czaja J, Schlaak JF, Lehir HA, Autscbach F, Schurmann G, Nishimoto N, Yoshizaki K, Ito H, Kishimoto T, Galle PR, Rose-John S, Neurath MF (2000). Blockade of interleukin-6 trans-signaling suppresses T cell resistance against apoptosis in chronic intestinal inflammation: evidence in Crohn's Disease and experimental colitis in-vivo. Nat Med.

[46] Rogacev KS, Cremers B, Zawada AM, Seiler S, Binder N, Ege P, Große-Dunker G, Heisel I, Hornof F, Jenken J, Rebling NM, Ulrich C, Scheller B, Böhm M, Fliser D, Heine GH (2012). CD14++CD16+ monocytes independently predict cardiovascular events: a cohort study of 951 patients referred for elective coronary angiography. J Am Coll Cardiol.

[47] Rogacev KS, Ulrich C, Blömer L, Hornof F, Oster K, Ziegelin M, Cremers B, Grenner Y, Geisel J, Schlitt A, Köhler H, Fliser D, Girndt M, Heine GH (2010). Monocyte heterogeneity in obesity and subclinical atherosclerosis. Eur Heart J.

[48] Rossol M, Kraus S, Pierer M, Baerwald C, Wagner U (2012). The CD14brightCD16+ Monocyte subset Is Expanded in Rheumatoid Arthritis and Promotes Expansion of the TH17 Cell Population. Arthritis Rheum.

