# Supplementary material for: COPD monocytes demonstrate impaired migratory ability
Source: Respir Res. 2017 May 11;18:90. doi: 10.1186/s12931-017-0569-y (PMC5425971; doi:10.1186/s12931-017-0569-y)
Supplement: Supplementary file 1 — Details of methodology (pulmonary function, plasma separation & cytokine measurement, flow cytometric monocyte characterisation, CD14+ monocyte isolation, sputum supernatant induced monocyte chemotaxis, CD14+ monocyte chemokine receptor gene expression, Quantitative polymerase chain reaction, Immunohistochemistry and Immunofluorescence. (DOCX 34 kb) [file 12931_2017_569_MOESM1_ESM.docx]

**Additional file 1 Methodology**

**Subjects**

Patients with a history of asthma, respiratory tract infection or COPD exacerbation within the preceding 6 weeks were excluded. The research was approved by the Greater Manchester (East) ethics committee: Approval numbers (10/H1003/108 & 05/Q140241)).

**Pulmonary function**

Maximum expiratory flow volume experiments were performed in triplicate on a Vitalograph gold standard wedge bellows spirometer; the highest FEV1 (forced expiratory volume in 1 second) and FVC (forced vital capacity) were recorded. Readings were repeated after 20 minutes following the inhalation of 200μg salbutamol via a spacer

**Exacerbation diagnosis**

A diagnosis of COPD exacerbation was made if 2 major symptoms (increased breathlessness, sputum purulence or sputum volume) or 1 major and 1 minor symptom (coryza, increased wheeze/chest tightness, sore throat, increased cough, fever) were reported as being present for a duration of 2 days.

**Plasma separation and cytokine measurement**

Following venesection, 10 mls of blood was collected into EDTA treated Vacutainer tubes. These tubes underwent centrifugation at 1500g for 15 minutes at room temperature. The supernatant was aspirated and stored frozen at -80°C until use.

Analyses were performed at UCB (Slough, UK). The lower limits of quantification (LLOQ) of the MSD assays were: IL-6 (1.6 pg/ml) and sIL-6R (0.1 pg/ml). Levels registering below the LLOQ were assigned a value of 0 pg/ml.

**Characterization of monocyte chemokine receptor expression**

10 mls of blood was obtained by antecubital venipuncture from stable state COPD patients and was collected into a heparinized container. Red blood cells (RBC) were lysed using a red cell lysis buffer (Biolegend, Cambridge UK). All cells were labelled with antibodies to CD14 (PE-Cy7), CD16 (APC), HLA-DR (APC-Cy7) in addition to a lineage specific cocktail (Per-CPCy5.5 conjugated anti-CD3, CD19 and CD56) to enable monocyte identification. Cell suspensions were also labelled with FITC conjugated anti-CCR5 antibodies. Antibodies were purchased from Biolegend, eBioscience (eBioscience, Hatfield UK) and BD (Becton-Dickenson, Oxford UK). Cells were incubated with these antibodies for 30 minutes in the dark. The flow cytometric assay was performed using a BD FACS Canto II flow cytometer (Becton-Dickenson) running BD FACS DIVA version 6 software. Colour compensation was performed to account for any spectral overlap between fluorophores. 1x10^6^ events were recorded. Cell doublets were removed by excluding those events demonstrating high FSC height and FSC width signals. Monocytes were identified using forward scatter and side scatter characteristics in addition to the expression or absence of particular surface antigens. Monocytes were identified amongst the HLA-DR+ cells that do not express CD3 (T lymphocyte), CD19 (B cell) or CD56 (natural killer cell). The three principal monocyte subtypes can be identified according to their relative expression of CD14 (LPS receptor) and CD16 (FcγRIII receptor): classical CD14^++^CD16^-^, intermediate CD14^+^CD16^+^ and non-classical CD14^-^CD16^++^. All procedures were performed at room temperature. Peripheral blood monocyte characterisation was also undertaken on COPD patients experiencing acute exacerbation.

**CD14^+^ monocyte isolation**

50 mL of blood was obtained by venepuncture and collected into a heparinized tube. Peripheral blood mononuclear cells (PBMC) were isolated by Ficoll differential centrifugation. CD14^+^ monocytes were positively collected from the PBMCs using magnetic bead isolation as previously described (1) and then suspended in PBS or RPMI (Sigma-Aldrich, Gillingham UK) (for chemotaxis and gene expression experiments respectively) at a concentration of 1x10^6^/mL.

**Sputum supernatant induced monocyte chemotaxis**

The chemotaxis assay was performed using 96-well Multiscreen MIC plates incorporating 5 μm pore diameter polycarbonate filter membranes (Merck-Millipore, Watford, UK) based on the method of transwell diffusion incorporating a fluorescence based detection method. CD14^+^ monocytes (1x10^5^ cells/well) were applied to the upper plate. For all experiments, sputum supernatant obtained from 3 individuals (either COPD patients or HNS) were pooled and diluted in a 1:10 ratio with PBS. Diluted sputum supernatant was applied to the wells of the lower plate. The two plates were incubated separately for 60 minutes at 37°C then sandwiched together and incubated for a further 18 hours. Migrated cells in the lower plate were collected, incubated with lysis buffer containing the Quant-IT PicoGreen fluorescent dsDNA binding probe (Life Technologies, Paisley UK) and analysed using a BMG LabTech FluoStar Optima optical density plate reader. CD14^+^ monocyte chemotaxis was expressed as a proportion (%) of maximum CD14^+^ monocyte migration (CD14^+^ cells added directly to the wells of the lower plate). All conditions were assayed in triplicate with mean chemotaxis values taken of technical replicates.

**CCL3 induced monocyte chemotaxis**

The chemotaxis assay was performed as described above however rhCCL3 (recombinant human) was utilised as the chemoattractant. Recombinant human CCL3 (R&D Systems, Abingdon, UK) was diluted in RPMI 1640 medium (without phenol red) (Sigma Aldrich, Irvine, UK) yielding concentrations of 250, 125, 62.5, 31.3, 15.6, 7.8, 3.9 and 2ng/ml.

**CD14^+^ monocyte chemokine receptor gene expression (cell culture)**

CD14^+^ monocytes were applied to the wells (4x10^5^ cells/well) of a 24-well plate (Greiner Bio-One, Stonehouse UK). All conditions were assayed in duplicate. Cell lysates were harvested using RLT buffer (with 1% β-mercaptoethanol) at 0h (unstimulated only), 4hrs and 19 hrs post-incubation.

**Quantitative polymerase chain reaction (QPCR)**

Total RNA was purified from cell lysates using RNeasy kits (Qiagen, Crawley-UK) according to the manufacturer’s instructions. DNA contamination was prevented by on column addition of DNase (Qiagen, Crawley – UK) according to manufacturer’s instructions. TaqMan reverse transcription PCR (RT-PCR) was performed on RNA from CD14^+^ monocytes (Thermo Scientific, Surrey-UK). Total RNA was then converted to cDNA. QPCR was performed using cDNA; 50ng of cDNA was used in 25μl reactions containing specific primers and probes for CCR5. Thermal cycling was performed using a Stratagene MX3005P (Agligent Technologies, West Lothian UK). Relative expression levels were determined using the ΔΔCt method normalizing to the endogenous control (GAPDH - Glyceraldehyde 3-phosphate dehydrogenase) with mean expression levels taken of technical replicates.

**Immunohistochemistry: Tissue preparation**

Explanted tissue was obtained from individuals undergoing surgical resection of lung carcinoma. Tissue was obtained from resected lung as far distal to the tumour as possible. Tissue was formalin fixed, embedded in paraffin and cut into 3-μm sections using a Leica RM microtome and lifted onto positively-charged adhesive glass slides (X-tra^®^, Leica Biosystems).

Tissue sections underwent dewaxing (xylene) and rehydration (immersion in descending concentrations of industrial methylated spirits: 100%, 90%, 75% and 50%) prior to immuno-staining. Heat induced epitope retrieval (HIER) was performed on rehydrated sections using pH 9.0 Tris EDTA buffer with Tween20 or pH 6.0 citrate buffer solutions.

**Immunohistochemistry**

Immunohistochemistry was performed using the ImmPRESS Excel Anti-mouse kits (Vector). These kits employ an enzymatic non-biotin amplification technique: primary antibodies were detected by secondary antibodies conjugated to the ImmPRESS Excel micropolymer. Sections initially underwent endogenous peroxidase blockade in Bloxall reagent for 10 minutes followed by blockade of non-specific antigen binding using 2.5% normal horse serum. Tissue sections were incubated with the mouse anti-human CD34 (Dako, Ely UK), neutrophil elastase (NE) or Ki67 primary antibodies for 30 minutes at room temperature. Amplifier antibody was then applied to each section. CD34, Ki67 and NE labelled cells were detected using horse anti-mouse secondary antibodies conjugated to ImmPRESS Excel Reagent and were visualised using 3,3’-diaminobenzidine substrate; counterstained with Gill’s haematoxylin and ‘blued’ using Scott’s tap water substitute. Following counterstaining, sections underwent dehydration in an ascending alcohol series (50%, 75%, 90%, 100% IMS) and clearing in xylene before being mounted with DPX (Sigma). Omission of the primary antibody from staining protocol was used a negative control.

**Immunofluorescence**

Specimens underwent pre-treatment (20 minutes at 800Watts) in pH 6.0 (0.01M) trisodium citrate buffer. Rabbit anti-human CX_3_CR1 (EMD Millipore, West Lothian UK) (diluted 1:800 in 1.5% normal donkey serum) was applied to the sections and left to incubate overnight at 4°C. This was detected using an Alexafluor 568 conjugated donkey anti-rabbit secondary antibody (Invitrogen, Paisley UK). Sections were next labelled with CD14/CD16 primary antibodies and detected using biotinylated secondary antibodies (see table 1); these were rendered fluorescent by application of Streptavidin Dylight 488 (Vector, Peterborough UK). A hard-set mountant containing the nuclear stain 4’, 6-diamidino-2-phenylindole (Vectashield Hardset mountant with DAPI, Vector) was used to mount the sections. Omission of primary antibodies was carried out on parallel sections enabling generation of control specimens.

**Table 1 Details of primary and secondary antibodies used for IF**

| **Primary antibody** | **Dilution factor** | **HIER buffer** | **Secondary antibody** |
| --- | --- | --- | --- |
| CD14  (Goat anti-human) | 1:100 | Trisodium citrate pH 6.0 | Biotinylated rabbit anti-goat IgG |
| CD16  (Mouse anti-human) | 1:50 | Trisodium citrate pH 6.0 | Biotinylated horse anti-mouse IgG |
| CX_3_CR1  (Rabbit anti-human) | 1:800 | Trisodium citrate pH 6.0 | Donkey anti-rabbit Alexa Fluor 568 |

**Table 1** This table shows primary antibodies, HIER buffer solutions and secondary antibodies. Secondary antibodies were diluted 1:200 with TBS (Tris-buffered saline) prior to application to the section.

**Imaging: Immunohistochemistry and Immunofluorescence**

Digital micrographs were obtained using a Nikon Eclipse 80i microscope (Nikon UK Ltd, Surrey UK) equipped with a QI imaging digital camera and Image Pro Plus 5.1 software (Media Cybernetics, Marlow UK). To obtain dual labelled images, fluorescent images from the same field were captured and digitally merged to determine CX_3_CR1^+^CD14^+^ and CX_3_CR1^+^CD16^+^ cells respectively.

**Image analysis-**

**Immunofluorescence**

Serial sections from each patient underwent dual labelled immunofluorescent staining for identification of CX_3_CR1^+^CD14^+^ and CX_3_CR1^+^CD16^+^ cells; CX3CR1 is a surface marker expressed by cells of a monocytic lineage (2). Five vessels were selected at random across each slide for imaging. For each patient, different vessels were selected and imaged in the serial sections stained with the CX_3_CR1^+^CD14^+^ and CX_3_CR1^+^CD16^+^ combinations; in total per 10 different vessels were imaged per patient. The proportion of marginated (apposition of the cell surface membrane to the luminal endothelial surface) CX_3_CR1^+^ (total 10 vessels), CX_3_CR1^+^CD14^+^ (5 vessels) and CX_3_CR1^+^CD16^+^ (5 vessels) cells (expressed as a percentage of all marginated cells) was quantified. To obtain dual-labelled fluorescent images, digital photomicrographs from the same field were captured and digitally merged to determine CX_3_CR1^+^CD14^+^ and CX_3_CR1^+^CD16^+^ cells. Digital micrographs were obtained through the use of a Nikon Eclipse 80i microscope (Nikon UK Ltd, Kingston upon Thames, UK) equipped with a QImaging digital camera and ImagePro Plus 5.1 software (Media Cybernetics, Marlow UK). Cell counts were quantified using the ImagePro Plus 5.1 software. Intravascular marginated cell counts were standardised to the number of positive cells per μm of vessel circumference. By standardizing marginated cell counts to vessel circumference; bias introduced by counting the number of marginated cells in vessels with large diameters is reduced (which may contain more marginated cells merely by the virtue of their size).

Microvessels were identified on the basis of their morphology (luminal erythrocyte content, endothelial cell monolayer); confirmatory immunohistochemical staining of the endothelial cell marker CD34 was performed on serial sections (3).

**Immunohistochemistry**

The entirety of the slide was imaged permitting determination of the overall area of the section. The overall number of Ki67^+^/Ki67^-^ alveolar macrophages (identified on the basis of their morphological characteristics) per section was determined and percentage positivity was calculated. AM counts were standardised to the number of positive cells per mm^2^ of the area of the section.

**References**

1. Ravi AK, Khurana S, Lemon J, Plumb J, Booth G, Healy L, Catley M, J V, Singh D. Increased levels of soluble interleukin-6 receptor and CCL3 in COPD sputum. *Respir Res* 2014; 15: 103.

2. Cros J, Cagnard N, Woollard K, Patey N, Zhang S-Y, Senechal B, Puel A, Biswas SK, Moshous D, Picard C, Jais J-P, D'Cruz D, Casanova J-L, Trouillet C, Geissmann F. Human CD14^dim^ Monocytes Patrol and Sense Nucleic acids and viruses via TLR7 and TLR8 receptors. *Immunity* 2010; 33: 375-386.

3. Pusztazeri MP, Seelentag W, Bosman FT. Immunohistochemical Expression of Endothelial Markers CD31, CD34, von Willebrand Factor, and Fli-1 in Normal Human Tissues. *J Histochem Cytochem* 2005; 54: 385-395.
