# Supplementary material for: COPD monocytes demonstrate impaired migratory ability
Source: Respir Res. 2017 May 11;18:90. doi: 10.1186/s12931-017-0569-y (PMC5425971; doi:10.1186/s12931-017-0569-y)
Supplement: Supplementary file 2 — Details of ‘Within-subject analysis’ (Generalised Estimation Equation analysis) from CCR5 gene expression study (DOCX 15 kb) [file 12931_2017_569_MOESM2_ESM.docx]

**Additional file 2: CCR5 gene expression ‘Within-subject analysis’**

**Statistical analysis**

Data from RT-PCR was expressed as fold-changes in gene expression above that of unstimulated control samples. Additionally, a ‘within-subject’ analysis (Generalised Estimation Equation (GEE) regression model) was conducted to compare the effects of stimulation (IL-6, IL-6+sIL-6R) and stimulation time (4h, 19h) on monocyte gene expression. This incorporated a pairwise comparison of conditions (stimulus) and culture duration (time). Individual values from duplicates were entered into the model.

**Results**

The within-subject analysis confirmed that the type and duration of cytokine stimulation were significant factors in the determination of CCR5 gene expression by CD14+ monocytes. Cells stimulated with IL-6+sIL-6R for 4 hours demonstrated significantly greater levels of CCR5 gene expression compared to basal unstimulated (mean difference 0.57, p<0.0001) and IL-6 stimulated cells (mean difference 0.51, p<0.0001).
