# Supplementary material for: COPD monocytes demonstrate impaired migratory ability
Source: Respir Res. 2017 May 11;18:90. doi: 10.1186/s12931-017-0569-y (PMC5425971; doi:10.1186/s12931-017-0569-y)
Supplement: Supplementary file 3 — Details of legends for additional Figures S1-S6. Table S1. demographic details of patients from cytokine analysis, flow cytometry, chemotaxis and gene expression studies. Table S2. proportions of peripheral blood monocyte subtypes (CD14++CD16-, CD14+CD16+ & CD14-CD16++) in stable and exacerbating COPD patients. Figure S1. proportions of monocyte sub-populations in the blood of COPD, S & HNS displayed as a graph. Figure S2. changes in the CCR5 expression by monocyte sub populations during COPD exacerbations displayed as a graph. Figure S3. Eeffect of age on CD14+ monocyte migration displayed as a graph. Figure S4. CD34 expression of pulmonary endothelial cells displayed as an immunohistochemistry image. Figure S5. neutrophils in the pulmonary microvasculature of COPD patients displayed as an immunohistochemistry image. Figure S6. Double negative immunofluorescent image of tonsilar tissue stained using an immunofluorescence protocol with omission of CX3CR1 and CD14 primary antibodies. (ZIP 2021 kb) [file 12931_2017_569_MOESM3_ESM.zip › Additional Table 2.docx]

**Additional Table 2**

|  | **CD14^++^CD16^-^ (%)** | **CD14^+^CD16^+^ (%)** | **CD14^-^CD16^++^ (%)** |
| --- | --- | --- | --- |
| **Stable** | 84.5 (2.3) | 7.9 (1.5) | 5 (1.0) |
| **Exacerbating** | 87.2 (5.1) | 9.1 (5.6) | 3.8 (1.2) |
| **p value** | ns | ns | ns |

**Additional Table 2** This table shows the proportions of CD14^++^CD16^-^, CD14^+^CD16^+^ and CD14^-^CD16^++^ monocytes in stable and exacerbating COPD patients. Data presented as mean (SD). The statistical significance of differences observed was determined by paired t-test. ns = not significant.
